# Centromeric Satellite DNAs: Hidden Sequence Variation in the Human Population

**DOI:** 10.3390/genes10050352

**Published:** 2019-05-08

**Authors:** Karen H. Miga

**Affiliations:** UC Santa Cruz Genomics Institute, University of California, Santa Cruz, California, CA 95064, USA; khmiga@soe.ucsc.edu; Tel.: +1-831-459-5232

**Keywords:** satellite DNA, centromere, sequence variation, structural variation, repeat, alpha satellite, human satellites, genome assembly

## Abstract

The central goal of medical genomics is to understand the inherited basis of sequence variation that underlies human physiology, evolution, and disease. Functional association studies currently ignore millions of bases that span each centromeric region and acrocentric short arm. These regions are enriched in long arrays of tandem repeats, or satellite DNAs, that are known to vary extensively in copy number and repeat structure in the human population. Satellite sequence variation in the human genome is often so large that it is detected cytogenetically, yet due to the lack of a reference assembly and informatics tools to measure this variability, contemporary high-resolution disease association studies are unable to detect causal variants in these regions. Nevertheless, recently uncovered associations between satellite DNA variation and human disease support that these regions present a substantial and biologically important fraction of human sequence variation. Therefore, there is a pressing and unmet need to detect and incorporate this uncharacterized sequence variation into broad studies of human evolution and medical genomics. Here I discuss the current knowledge of satellite DNA variation in the human genome, focusing on centromeric satellites and their potential implications for disease.

## 1. Introduction

Genome-scale initiatives, such as the Human Genome Project and the 1000 Genome (1KG) consortium [1,2,3], have provided a wealth of genomic information that have greatly advanced basic and biomedical research. However, in light of this progress, the millions of bases that span each human centromeric region remain largely disconnected from contemporary genetic and genomic analyses. This has historically been due to the challenge of generating and validating linear assemblies of tandemly-repeated DNA (e.g., thousands of copies of a repeat with a limited number of sequence variants to guide overlap-consensus derived assemblies), which are known to span each centromeric region [4]. Our understanding of the sequence content and organization of human centromeres improved dramatically with the release of the GRCh38 reference genome, and recent efforts to generate true linear assemblies using “ultra-long” sequencing (i.e., reads that span hundreds of kilobases [5]), wherein the centromere-assigned gaps on each chromosome assembly were updated with sequence information [6,7]. Thus, we are entering a new era in genomics where centromeric DNAs are available for detailed study, either within a single karyotype or across human populations, that will drive research aimed to understand repeat variation that contributes to genome stability, population variation, and disease. 

Centromeric satellite DNA arrays are known to vary extensively in the human population, yet few genomic tools have been developed to study the full extent of this sequence variation, thereby ignoring a fraction of the human genome expected to contribute directly to cancer and human disease [8,9,10]. The extent of variation has been documented at the cytogenetic level, and gross estimates of rearrangement and/or repeat expansion have been associated with cancer and infertility [11,12,13]. Additionally, the epigenetic regulation of satellite DNAs, as well as anomalous methylation and altered transcription of satellite DNAs, have been associated with human diseases [9,14]. However, these early observations are challenged by inconsistencies in association [15], small sample size and perhaps an incomplete (or often, low-resolution) understanding of underlying genomic structure and array variant composition. 

Acknowledging the differences between the satellite arrays, there is limited utility in restricting studies to the use of a single genomic map. Rather, it is important to extend our survey of satellite DNA genomics to large panels of diverse individuals, thus enabling high-resolution maps of human sequence diversity in these regions. Such sampling efforts would be the foundation for a modern era of satellite DNA genomics: establishing allelic frequencies for satellite variants necessary to expand disease-association studies. Here I discuss our current understanding of satellite DNA variation as determined from whole-genome sequencing projects, with a focus on the largest families in the human genome and their association with disease.

## 2. What Proportion of the Human Genome is Defined by Peri/Centromeric Satellite DNAs?

The largest arrays of satellite DNAs in the human genome are organized within centromeric and pericentromeric regions [2,3,16]. Although several distinct satellite DNA families are known to contribute to pericentromeric regions (e.g., gamma, beta, and subtelomeric satellites [17,18,19]), this review is focused on alpha satellite and human satellites 2, 3, which are the most abundant in the human genome and most commonly associated with human disease [8]. The alpha satellite DNA family is defined by a group of related, highly divergent AT-rich repeats or ‘monomers’, each approximately 171 bp in length, which are found in every normal human centromere [20,21,22]. Previous genome-wide estimates of alpha satellite have observed that this family represents ~2.6% of the human genome [23], which roughly aligns with early hybridization-based estimates [16]. Additionally, previous physical maps of centromeric regions, and pulse-field gel electrophoresis (PFGE) southern-based estimates of chromosome-assigned satellite arrays, revealed an average (~3 Mbps) amount of alpha satellite per centromeric region [24,25,26]. Therefore, we can assign a very rough estimate of 72 Mbps across all 22 autosomes and two sex chromosomes (i.e., 2.4%), which remains in agreement with all previous genome-wide estimates. Human satellites 2, 3 (HSat2,3), are collectively defined by enrichment of a pentameric repeat, (CATTC)n, and represent the largest heterochromatin blocks (documented as at least 10 Mbps in length) in human pericentromeric regions; notably, on chromosomes 1, 9, 16, and Y [27,28,29,30,31,32]. In total, HSat2,3 are observed to be less abundant than alpha satellite (~1.5% of the genome) [31], yet early estimates of array lengths on the DYZ1 array suggest that abundance estimates may vary considerably in the human population [31,33,34,35]. Therefore, efforts to better understand the true extent of satellite subfamily overall variation would benefit from surveying a much larger panel of diverse individuals. In doing so, one can define the lower and upper bounds of satellite DNA content in the genome. For example, can one individual have 3% alpha satellite and another individual have closer to 10%? What defines these bounds? Further, do these fluctuations in overall satellite composition contribute to our understanding of chromosome segregation, genome stability, and disease?

**Figure 1:**
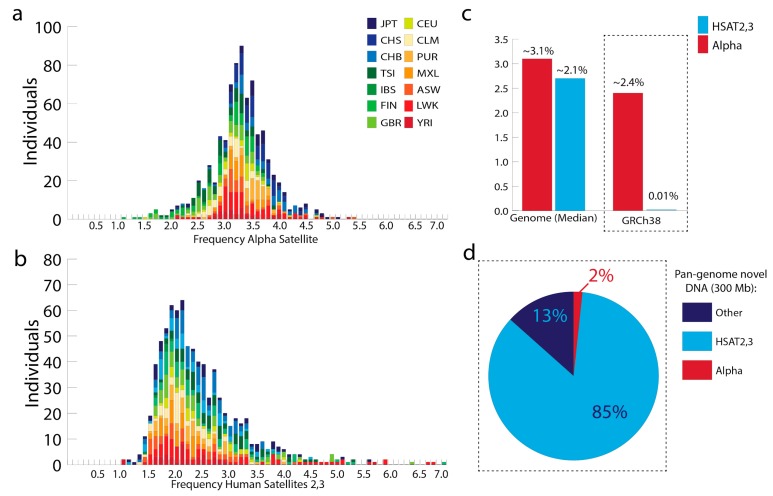
Proportion of alpha satellite and human satellites 2,3 in the human population. Using 1KG [1] data representing 14 diverse populations (400 male individuals and 414 female individuals) (a) the frequency of 24-mers that have an exact match with alpha satellite [23], (b) the frequency of 24-mers that have an exact match with human satellite 2,3 [31]. (c) Median frequencies (from panel (a) alpha and (b) HSat2,3) are listed relative to the observed frequency in the human reference genome assembly (GRCh38; GCA_000001405.15). (d) Evaluation of 300 Mb of DNA from the collective genomes of 910 people of African descent, previously determined to be missing or unaligned to GRCh38 [39]. Key for human subpopulations: CHB: Han Chinese in Beijing, China; JPT: Japanese in Tokyo, Japan; CHS: Southern Han Chinese; CEU: Utah Residents (CEPH) with Northern and Western European Ancestry; TSI: Toscani in Italia; FIN: Finnish in Finland; GBR: British in England and Scotland; IBS: Iberian Population in Spain; YRI: Yoruba in Ibadan, Nigeria; LWK: Luhya in Webuye, Kenya; GWD: Gambian in Western Divisions in the Gambia; ASW: Americans of African Ancestry in SW USA; MXL: Mexican Ancestry from Los Angeles USA; PUR: Puerto Ricans from Puerto Rico; CLM: Colombians from Medellin, Colombia.

Genome-wide estimates of alpha satellite and HSat2,3 content have relied on constructing comprehensive sequence databases using raw read data from each satellite DNA family, thereby avoiding underestimates due to assembly collapse of identical/near-identical repeats [23,31]. Alpha satellite and HSat2,3 exhibit considerable sequence heterogeneity [20,21,32], as observed most readily in the ability to hybridize specifically to divergent repeat sequences within chromosome-specific arrays [20,36,37,38]. Therefore, efforts to construct genome-wide libraries from short-read datasets rely on methods that are comprehensive and inclusive with respect to the potential heterogeneity within satellite families. One approach is to reformat published satellite sequence libraries [7,23,31] into catalogs of short oligonucleotide sequences (24 bps, representing sequences in both orientations) that are specific to a given satellite family; that is, each oligo is only observed within a respective satellite database and never observed to have an exact match anywhere else in the genome. It is then possible to survey existing low-coverage, publicly available, population datasets, such as 1KG data [1], to identify what percentage of reads (as determined by exact matches with oligo libraries) are assigned to each respective satellite family (Figure 1a,b). In a study of low-coverage 1KG sequence data representing 14 diverse populations (400 male individuals and 414 female individuals) [1,7], alpha satellite has a median of 3.1% genome-wide estimate, with a range between 1% and 5% (Figure 1a). These initial HSat2,3 estimates reveal that although this satellite family is typically less abundant than alpha (median, 2.1%, range ~1–7%), it is observed in many cases to match, or surpass, alpha satellite abundance [31] (Figure 1b).

Proper representation of satellite DNAs in human reference assemblies will be critical to ensure faithful short read mapping and accurate assessments of satellite family variability in the future. Notably, the addition of millions of bases of alpha satellite “reference models” with the release of the GRCh38 reference genome has provided initial short read mapping targets [7,40]. This has proven to be useful in decreasing off-target alignments and has enabled high-resolution studies aimed to study the epigenetic structure in centromeres [41,42]. In contrast, HSat2,3 are woefully underrepresented in all human assemblies, with only ~0.01% representation in GRCh38 (Figure 1c). This can lead to pronounced differences in the way we annotate and study human variation. For example, a recent study of 910 individuals of African descent identified roughly 300 Mbp of sequences not present in the human reference (GRCh38) [39], of which the largest proportion have exact oligo matches with HSat2,3 (Figure 1d). This demonstrates the importance of proper satellite DNA representation in the reference assembly in shaping our interpretation of novel sequences in the population. Notably, alpha satellite, a satellite family with great representation in the current reference assembly, still has candidate sequences that are not aligned to the reference models derived from the HuRef genome (Figure 1d, red). This result emphasizes a second important point: because satellites are expected to vary between genomes, the use of a single individual’s genome as a reference, in this case HuRef [43], is not sufficient to capture the sequence diversity in the human population. Expanding the representation of human sequence diversity has been previously shown to improve mapping of variants [44,45], highlighting the need for a ‘pan-human genome reference’ to improve mapping efficiency and satellite variation studies in the future. 

## 3. What is the Nature of Sequence Variation within a Single Satellite Array? 

The variation of satellite DNAs genome-wide abundance is driven by repeat expansion and contraction, commonly attributed to mechanisms of non-homologous crossover and/or conversion [46,47]. Genomic-based studies of satellite DNA evolution have greatly benefited from the advancement of software designed to study tandem repeat variation in unassembled reads (reviewed [48]). The advancement of such high-resolution studies across a broad number of species is expected to dramatically advance our knowledge of the rates and mechanisms driving satellite array evolution. Previous studies of comprehensive studies of satellite DNA classes. Efforts in the past to study satellite repeat variation have focused on shorter microsatellites and tandem repeat classes that are amenable to complete assembly using long read technologies. However, recent efforts to study tandem repeat variation in human rDNA arrays revealed a high level of heterogeneity (i.e., an average rate of 7.5 variants per kb). Each rDNA unit is 45 kb with roughly 500 copies per diploid cell, and much like the satellite arrays, rDNA array length can vary significantly in size from just a few units to >100 between individuals [49,50].

**Figure 2:**
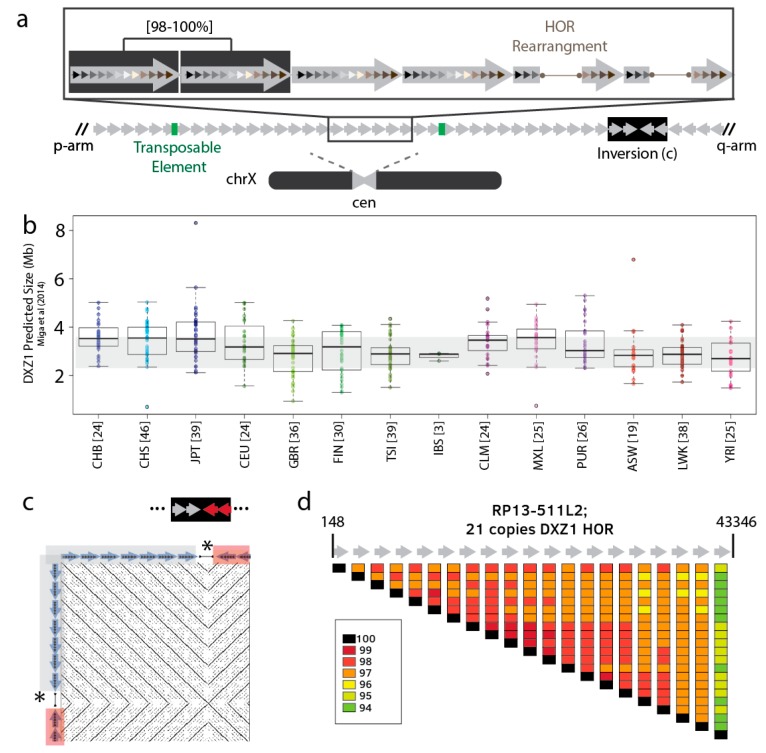
Intra-array satellite sequence variation. (a) All normal human centromeric regions contain at least one alpha satellite array, shown in grey, which is tandemly organized in a head-to-tail orientation with occasionally transposable element interruptions (green) and shifts in directionality (black box). The fundamental alpha satellite repeat unit, or ~171 bp monomer, is shown in a variation of shaded colors to illustrate the heterogeneity of the sequencing identity. Multi-monomer repeat units, or ‘higher-order repeats (HORs), are shown by the larger grey arrows that encompass the collection of smaller repeats. In contrast to the individual monomers, these repeats are shown to be identical, or near-identical (98–100%). In addition to single nucleotide differences between the HORs, larger rearrangements (shown as a deletion of five monomers) are observed to occur and expand and contract within the array. (b) Satellite array length predictions on the X chromosome (DXZ1) [7], grey shading marks the previously observed PFGE Southern length range [25]. (c) Inversion detected using error-corrected PacBio reads [68]. (d) RP13-511L2 is an X-specific BAC that represents the transition from core alpha satellite to the edge of the array. HOR pair-wise repeat identity (muscle alignment [72]) showing increased divergence approaching the chromosome arm (43,346 bp), as typically observed at the edge of the array.

The relationship between repeat units from different alpha satellite arrays would suggest that the rate of intra-chromosomal exchange (i.e., sister chromatid exchange) is higher than inter-homologue exchange [51]. As a result, most satellite arrays in the human genome can be defined by highly homogeneous arrays that can be often typified by chromosome-specific multi-monomeric repeat units, or higher-order repeats (HOR) [20,52]. Although the chromosome-assignment of HORs is largely invariant between individuals, as demonstrated by the effectiveness of commercially available satellite Fluorescence in situ hybridization (FISH) markers for chromosome labeling in clinical cytogenetics, the thousands of copies of the HOR that comprise a single array are expected to represent a mixture of expansion/contraction of repeat variants, shifts in orientation, and mobile element insertions (Figure 2a) [7,20,37,53].

This ever-changing genomic landscape guides our understanding of centromere function and chromosome stability. For example, the repeat structure and array length are expected to change the frequency of and spacing of the 17-bp centromere protein B binding motif, or CENP-B box. The functional role of CENP-B at human centromeres is not yet fully understood [54,55], yet recent studies suggest that the periodicity may contribute to kinetochore function and centromere fidelity [56,57]. Rearrangement in canonical HOR units, i.e., insertions and/or deletions presumably due to unequal crossing-over events, are observed at different frequencies between spatially distinct arrays. The Chr17-specific alpha satellite HOR (D17Z1) is characterized by arrays containing approximately 1000 repeat units that range in length from 11–16 monomers [36,56,58]. The frequency and ordering of these variants have been shown to influence the centromere location on human chromosomes with metastable epialleles [59,60]. Ultimately, sequence composition within each satellite array is thought to influence expression of the repeats [10,61,62], transcription factor binding [63,64], and replication efficiency [65,66,67]. Therefore, the high-resolution and comprehensive study of array sequence composition and structure is key to our understanding of how these specialized loci function.

Previous methods have used unassembled reads from whole-genome sequencing projects to evaluate chromosome-specific satellite overall abundance, or copy number, and the frequency of variants (e.g., HOR rearrangements, inversions, transposition, and single nucleotide variants (SNVs)) within the array [7,31,68]. Specifically, the centromeric regions on the X and Y chromosomes in male genomes offer a unique opportunity to study the variation in haploid array length within the human population. Altemose et al. [31], estimated the array size using low-coverage, short-read sequencing data from 396 male 1KG individuals [1], showing that the DYZ1 array varies over an order of magnitude (7–98 Mbps, with a mean of 24 Mb), consistent with previous experimental observations of Y-chromosome length variability [34,69,70]. Similarly, 1KG read-depth-based estimates of alpha satellite array lengths on the X and Y chromosomes (DXZ1 and DYZ3) agree with prior PFGE Southern experiments [7,25,71]. Although the X array has been predicted to have a 10-fold size range (800 kb to 8 Mbps), the medians of predicted X array lengths per human population, are observed to fall within experimentally validated lengths of 2.2–3.7 Mbps (mean 3010 kb) [7,25] (Figure 2b). This further corroborates the accuracy of short-read-based array length estimates applied to diverse groups of people.

Use of error-corrected long reads (e.g., Pacific Biosciences, PacBio) prior to assembly provide an automated method to identify larger structural variation (SV) in satellite arrays, such as: HOR rearrangement (insertion and/or deletion), inversions, and interruption by transposons [68]. In addition to changes in the HOR structure, one can monitor precise sites where shifts in orientation or inversions take place within the array (Figure 2c). Further, when tracking sites of transposable element insertion, LINE1 is documented to be the most prevalent, consistent with the literature of alpha satellite DNA [73]. In addition to advancing our understanding of sequence organization and centromere function, such low-copy sequence variants that interrupt the uniformity of the satellite array are also expected to also guide linear assembly efforts [4,6]. Likewise, low-copy SNVs have been shown to be useful in overlap-consensus assembly, but they depend on high sequencing accuracy often obtained from Illumina reads and/or high-coverage of long-read data [6]. Satellite DNA studies using bacterial artificial chromosome (BAC) data provide a snapshot of local SNV spacing, where increased divergence is expected at the edges of the array (closest to the transition with the chromosome arms) with sparse, and infrequent informative sites within the array (Figure 2d) [73]. Ultimately, efforts to construct robust databases of satellite-associated SVs and SNVs will benefit from additional high-coverage, long read (PacBio or nanopore sequencing) datasets from diverse individuals. Such databases would provide allele-frequency data needed to guide future disease associations of variants.

## 4. Centromeric Regions Span Variants Associated with Disease. 

**Figure 3:**
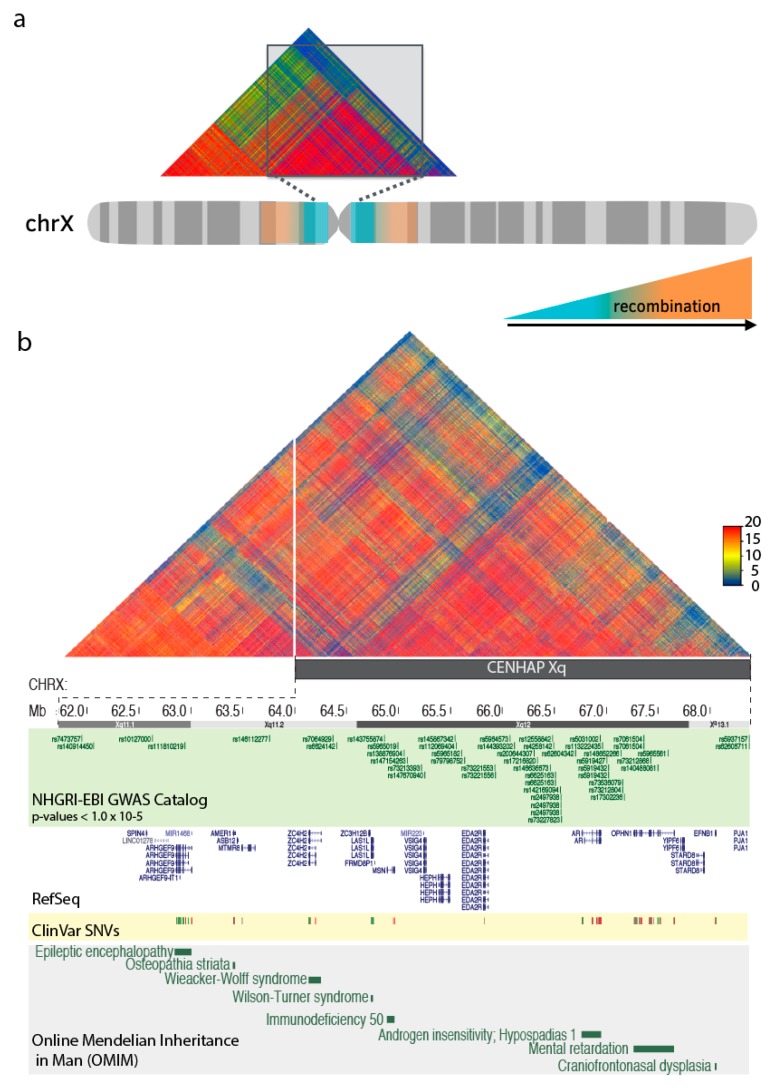
Disease-associated variants in centromere-associated haplotypes. (a) Centromeres act as the primary constriction of chromosomes, and are historically defined by the reduction of meiotic recombination (indicated by blue). Therefore, sequences in these regions are commonly inherited in large linkage blocks, or cenhaps (shown in the linkage disequilibrium heat map) [70]. (b) Study of disease and clinically associated single nucleotide variants (GWAS Catalog (green), ClinVar SNVs (yellow) in the Xq cenhap region (with Linkage Disequilibrium heat map from (a) enlarged) and a collection of annotated genes (RefSeq, white), of which variation have been attributed to a human disease (OMIM data, grey).

Entire multi-megabase-sized centromeric regions, including the heterochromatic regions in the pericentromere, suppress meiotic recombination and are commonly observed as a single haplotype block, or ‘cenhap’ (Figure 3a) [74]. Little is known about the unique evolution and regulatory properties of those sequences that are associated with these highly specialized regions. Position effect variegation (PEV), or the mosaic pattern of gene expression when placed within or near heterochromatic environments, has been observed in organisms from yeast to humans [71]. The extensive range of satellite array sizes observed within the human population may contribute to studies of PEV variability and gene regulation in the human genome. Sequences directly adjacent to the centromeric satellite arrays have been documented as hypermutable, with a speculation that the increased mutation rate may be attributed to centromere activity [73,74]. Further, genes that are largely excluded by recombination may influence the efficacy of selection and create a ‘protected’ environment for gene mutations, inheritance, and disease. 

These immense linkage blocks encompass satellite DNAs, segmental duplications [75], and a collection of well-annotated genes [76], many of which have been previously attributed to human clinical and disease phenotypes. Although the functional implications of gene-level associations are difficult to infer due to the large region of linkage disequilibrium, it may be useful for studies to recognize and bin these centromere-associated genomic regions as it is likely that they are share a compartment of the genome with specialized inheritance and evolution. The Xq cenhap region contains eight genes that are documented in the Online Mendelian Inheritance in Man (OMIM) noting the potential for allelic variants to represent disease-causing mutations (Figure 3b) [77,78]. Additionally, genome-wide association studies (GWAS) have identified SNPs in cenhap regions as associated with human disease (as shown in Figure 3b for NHGRI-EBI GWAS data, each selected with p-values < 1.0 × 10–5), many of which do not overlap with genes or annotated sequence features [79,80]. Studies of variants directly adjacent to centromeric regions have been associated with chromosome instability and disease. For example, multiple independent signals associated with chromosome X loss around the centromere of chromosome X have been reported in a study of mosaic chromosomal alterations in clonal hematopoiesis [81], with a strong association (P = 6.6 × 10−27, with an observed 1.9:1 bias in the lost haplotype) near the centromere array (DXZ1, Xp11.1). Further examples of centromere-adjacent or associated SNPs have been used to predict a significant association with multiple sclerosis risk around the chromosome 1 (lod = 4.9; with initial scan of 484 cases and 1043 controls; genotyped at 1082 SNPs) [82]. It is likely that many other disease association loci exist in these centromere-proximal regions, as association with centromeric SNPs (defined as within 2 Mbps of an alpha satellite reference model in GRCh38 that do not overlap with a known gene or segmental duplication) have been observed for a variety of clinical studies, including various cancers, neurodegenerative disorders and cardiovascular diseases (Table 1) [80,83].

In addition to studies that involve the cenhap associated regions, extensive sequence variation within the satellite array is expected to contribute to our understanding of centromere instability and disease. Cytogenetic staining has revealed the constitutive heterochromatin in human centromeric regions has a highly heteromorphic structure. Given the critical importance of centromeres in ensuring proper chromosome segregation, such genomic variation is hypothesized to drive genome stability, and have been linked with human disease and cancers (reviewed [84]). Nevertheless, in the case of cancers where cells are expected to present increased genomic rearrangements, altered regulation and localization of kinetochore proteins, it will be important to estimate the rate of neocentromere formation with respect to native centromere sequence stability to test functionally relevant satellite DNA variants. We are only beginning to understand the sequence organization, allelic frequency, and evolution of satellite DNAs in the human population. Indeed, an analysis of optical genome maps of 154 individuals from 26 populations provided evidence for a large proportion of structural variants in satellite DNAs [85]. Such high-resolution diversity maps are expected to guide studies aimed to characterize satellite array structures that are associated with disease from those that have little functional consequence.

## 5. Concluding Remarks

In conclusion, human satellite DNAs provide a new, largely uncharted source of sequence variation in the human population. Chromosome-specific satellite arrays are expected to vary considerably in the human population, and measuring the overall range in the abundance and frequencies of repeat variants will contribute to ongoing studies of centromere biology and genome instability. Efforts to identify and study these variants will rely on improved, comprehensive genomic methods capable of mapping the full extent of satellite sequence heterogeneity that cannot be captured using a single reference genome. Such maps are necessary to direct future biomedical research to variants that are associated with disease, rather than natural sequence variation, which may have little or no clinical consequence.

## Figures and Tables

**Table 1:** Description of centromere-adjacent single nucleotide polymorphisms (SNPs) identified by published Genome-Wide Association Studies (GWAS), collected in the NHGRI-EBI GWAS Catalog published jointly by the National Human Genome Research Institute (NHGRI) and the European Bioinformatics Institute (EMBL-EBI) [80]. SNPs are included if found within a two-megabase window of an alpha satellite reference model (GRCh38) and do not overlap with annotated genes or segmental duplication).

Trait	SNPs	CEN adjacent (2Mb) Regions	Citation
Cancer	rs930395, rs2241024, rs142427110, rs35951924, rs199501877, rs11146838, rs6490525, rs2050203, rs7278690, rs35505947	4p12; 5p12; 5q11; 10p11; 13q12; 18p11; 19q11; 20p11; 21q11	[86,87,88,89]
Cardiovascular disease	rs10132760, rs12186641, rs9367716, rs71566846, rs223290, rs144961578, rs3813127, rs1657346, rs1254531, rs10793514	5q11.2; 6p11.2; 6q11.1; 10q11.21; 14q11.2; 18q11.2	[90,91,92]
Neurodegenerative diseases	rs11826064, rs13168838, rs62365447, rs140996952, rs1480597, rs10783624, rs7989524, rs6822736, rs13110633, rs2424635	4p11; 4q12; 5p12; 5q11.1; 6q11.1; 10q11; 11p11; 12q12; 13q12; 20p11	[93,94,95,96,97,98,99,100]
Scoliosis/Bone Density (Spine)	rs8111296, rs11652527, rs1436931, rs6061081, rs17599071, rs10136383, rs9288898, rs10772040, rs4562194, rs810967, rs6050182, rs6511621, rs11229654, rs6551418, rs1006899	3p11.1; 3q11.2; 6q12; 7q11.21; 10q11.21; 11q11; 12p11.21; 14q11.2; 17q11.2; 19p12; 20p11.21; 21q11.2	[101,102]
Digestive system disease	rs4243971, rs2342002, rs4800353, rs6058869, rs6087990	6q11.1; 18q11.2; 20q11.21	[103,104]
